# Guard Cell-Specific Pectin METHYLESTERASE53 Is Required for Abscisic Acid-Mediated Stomatal Function and Heat Response in *Arabidopsis*

**DOI:** 10.3389/fpls.2022.836151

**Published:** 2022-02-21

**Authors:** Hui-Chen Wu, Shih-Yu Yu, Yin-Da Wang, Tsung-Luo Jinn

**Affiliations:** ^1^Department of Life Science, Institute of Plant Biology, National Taiwan University, Taipei, Taiwan; ^2^Department of Biological Sciences and Technology, National University of Tainan, Tainan, Taiwan

**Keywords:** ABA response, guard cell, pectin, pectin methylesterase, stomatal-lineage pathway, thermotolerance

## Abstract

Pectin is a major component of the plant cell wall, forming a network that contributes to cell wall integrity and flexibility. Pectin methylesterase (PME) catalyzes the removal of methylester groups from the homogalacturonan backbone, the most abundant pectic polymer, and contributes to intercellular adhesion during plant development and different environmental stimuli stress. In this study, we identified and characterized an Arabidopsis type-II *PME*, *PME53*, which encodes a cell wall deposited protein and may be involved in the stomatal lineage pathway and stomatal functions. We demonstrated that *PME53* is expressed explicitly in guard cells as an abscisic acid (ABA)-regulated gene required for stomatal movement and thermotolerance. The expression of *PME53* is significantly affected by the stomatal differentiation factors SCRM and MUTE. The null mutation in *PME53* results in a significant increase in stomatal number and susceptibility to ABA-induced stomatal closure. During heat stress, the *pme53* mutant highly altered the activity of PME and significantly lowered the expression level of the calmodulin *AtCaM3*, indicating that PME53 may be involved in Ca^2+^-pectate reconstitution to render plant thermotolerance. Here, we present evidence that the PME53-mediated de-methylesterification status of pectin is directed toward stomatal development, movement, and regulation of the flexibility of the guard cell wall required for the heat response.

## Introduction

Plants are sessile organisms that have to cope with the challenge of extreme environmental conditions, including various abiotic and biotic stresses, all of which exert adverse effects on plant growth and development. The most likely negative impact of these changes is an increase in temperature, which directly impacts DNA, proteins, cell membranes, and metabolism processes, severely limiting plant growth, development, and productivity. As a result, plants have devised a variety of strategies to deal with temperature extremes. Plant thermotolerance entails converting a moderate temperature increase into molecular defenses against subsequent extreme temperatures and preventing and repairing heat-labile protein and membrane damage (Larkindale and Vierling, 2008). The induction of molecular chaperone heat shock proteins (HSPs) is an essential part of the universally conserved heat response, allowing organisms to survive stress conditions such as elevated temperatures that cause protein unfolding (Lindquist and Craig, 1988).

Apart from the well-known heat stress response, little is known about how the dynamics of plant cell wall changes in wall composition and architecture are associated with various stresses. The plant cell wall is a sophisticated structure formed by a complex mixture of polysaccharide-rich proteins and other polymers assembled into a rigid, flexible, and dynamically organized network (Wolf et al., 2009; Wu et al., 2018). When cells are stressed, specific transcriptional responses, some profitable cell wall proteins, and critical changes in cell wall architecture are affected (Klis et al., 2006). In plant cells, the integrity of the cell wall is tightly controlled and closely coordinated with the stress response. We previously discussed key components of cell wall reconstruction during heat stress, such as pectin, pectin methylesterase (PME), and apoplastic Ca^2+^ (Wu and Jinn, 2010; Wu et al., 2010, 2018). Therefore, the maintenance of cell wall integrity is essential for cell viability and function during stress (Hamann et al., 2009; Wu et al., 2018).

Pectin is the most complex polysaccharide in the cell wall, which is synthesized and methylesterified in the Golgi and then secreted into the cell wall in a highly methylesterified state. Pectin is important for both cellular adhesion and cell wall plasticity, thus controlling cell and organ growth during plant development (Willats et al., 2001; Palin and Geitmann, 2012). Homogalacturonan (HGA), a major component of pectin, consists of linear α-1,4-linked D-galacturonic acid (D-GalA) residues and has conformational flexibility, which can be influenced by growth, development, and environmental cues (Willats et al., 2001). A critical feature of HGA that influences its properties is the methylesterification of C6 and acetylesterification on C2 or C3, which occurs on GalA during the synthesis of the backbone.

Subsequently, highly esterified HGAs exported into cell walls were de-methylesterified by the mature pectin methylesterases (EC 3.1.1.11; PMEs), which belong to class 8 (CE8) of the carbohydrate esterases (CAZy website)^1^ (Cantarel et al., 2009), which converts methoxyl groups into carboxyl groups on the HGA backbone, resulting in the release of methanol and protons. Plant PMEs belong to a multigene family whose members may play role in a broad range of physiological processes. For instance, 66 *PME*-related genes are predicted in *Arabidopsis thaliana*, 89 in *Populus trichocarpa*, 81 in *Gossypium raimondii*, 79 in *Lycopersicon esculentum*, 105 in *Linum usitatissimum*, 43 in *Oryza sativa* and *Zea mays* (Louvet et al., 2006; Liu et al., 2013; Pinzón-Latorre and Deyholos, 2013; Horowitz and Ospina-Giraldo, 2015; Jeong et al., 2015; Zhang et al., 2019). The precise biological functions of PMEs remain unclear although various roles have been proposed.

In higher plants, the *PME* gene encodes the individual PRE-PRO-region, which is considered a signature of PMEs. PMEs are led to the wall by the PRE domain, and the conserved PME domain, which is the active part of the protein, is preceded by the PRO region, which shares similarities with PME inhibitors (PMEIs) (Pelloux et al., 2007). Therefore, PMEs can be based on the presence or absence of the PRO region and are classified into type I and type II. Type I PMEs contain PRO regions, whereas type II PMEs do not. The type II PMEs sequences are similar to those of phytopathogenic organisms (fungi and bacteria) and are associated with cell wall softening and loosening during pathogen infection (Micheli, 2001).

The fine control of the degree of methylesterification is a key factor in HGAs and is potentially involved in the regulation of cell wall architecture, which determines that the methylesterification status of pectin is regulated by the action of PMEs. In recent years, PMEs have attracted public attention because of their roles in many aspects. Over the past few years, only several specific PME genes have been described and have revealed multiple roles in Arabidopsis. For example, overexpression of Arabidopsis *PME5* and *PMEI3* resulted in softer and harder shoot apical meristem cell walls, respectively, when compared with the wild-type plants (Peaucelle et al., 2011). Arabidopsis *PME35* is explicitly expressed in the basal part of the inflorescence stem and provides mechanical support (Hongo et al., 2012). Highly methyl esterified seeds (HMS; PME6) is abundant during mucilage secretion, acting on embryo morphology, and mucilage extrusion, both of which are involved in embryo development (Levesque-Tremblay et al., 2015). In stress responses, Arabidopsis PME41 and PME48 are associated with chilling/freezing tolerance through the regulation of brassinosteroid signaling and pollen grain germination, respectively (Qu et al., 2011; Leroux et al., 2015). The cyst nematode (*Heterodera schachtii*) parasitism can be reduced by inhibiting Arabidopsis PME3 to interact with the cellulose-binding protein of the cyst nematode (Hewezi et al., 2008). PME17 in Arabidopsis performs a linear (blockwise) de-methylesterification that promotes the formation of egg-box structures, which aids in *Botrytis cinerea* resistance (Del Corpo et al., 2020).

Stomata are pores on the surface of leaves, formed by a pair of curved guard cells with unique cell wall compositions that regulate gas and water exchange by opening and closing the stomata. The opening and closure of stomata pores are mediated by changes in the turgor pressure of the two guard cells, which can be affected by various signals, including CO_2_, humidity, red/blue light, abscisic acid (ABA), auxins, calcium (Ca^2+^), and extracellular calmodulin (Jones et al., 2003; Hõrak et al., 2017). In addition, guard cells have remarkable elasticity and can act reversibly during stomatal opening and closing due to differential thickening and the orientation of cellulose microfibrils. The shape of stomata is limited by the mechanical properties of cells, which largely depend on the structure and composition of cell walls that allow them to undergo repeated swelling and deflation. Although it has been known that the anisotropic nature of guard cell walls, showing differential wall thickness and the orientation of cellulose microfibrils determined the stomatal opening and closure, the role of cell wall mechanical properties remains an open question.

In Arabidopsis, the walls of guard cells are rich in un-methylesterified pectin, but the high-methylesterified HGA and Ca^2+^ crosslinked de-methylesterified HGA are absent from guard cell walls (Amsbury et al., 2016). This suggests that the stomatal function of guard cells may be influenced by the cell wall properties of guard cells. PME also appears to play a role in the regulation of plant development by influencing the mechanical properties of plant cell walls, according to some studies. The guard cell wall of Arabidopsis *pme6* mutant, for example, was rich in methylesterified pectin and had a narrower dynamic range in response to stomatal opening/closure triggers, implying that stomatal function loss is due to a mechanical change in the guard cell wall (Amsbury et al., 2016). Therefore, the function of stomata requires pectin de-methylesterification of guard cell walls through the regulation of PMEs. In our previous study, we identified PME34 which plays a role in regulating guard cell wall flexibility to control stomatal aperture involved in mediating heat response in Arabidopsis (Huang et al., 2017; Wu et al., 2017). Therefore, changes in cell wall metabolism and cell wall-modifying enzyme activity in controlling guard cell wall plasticity are important physiological mechanisms of plants to heat stress (Wu et al., 2017, 2018).

Guard cells can inflate and deflate repeatedly, and the formation of guard cell pores necessitates the modification of pectin. It causes pore initiation and enlargement by de-methylesterification of HGA (Rui et al., 2019). Although PME6 and PME34 have been shown to play a role in stomatal movement (Amsbury et al., 2016; Huang et al., 2017), little is known about how pectin modification affects stomatal development and downstream signaling pathways. It is known that stomatal development is regulated by a group of basic helix-loop-helix (bHLH) transcription factors, including SPEECHLESS (SPCH), MUTE, and FAMA, which act together and are critical in mediating guard cell differentiation at different steps of the stomatal lineage in Arabidopsis (Davies and Bergmann, 2014). Despite extensive investigations of the bHLH factors involved in stomatal development, the correlation between the mechanical properties of stomatal guard cells and the regulation of bHLH factors remains elusive.

In a previous study, we verified that maintaining apoplastic Ca^2+^ homeostasis through PME activity has a pronounced effect on plant growth and heat response (Wu et al., 2010). Using a genetic approach, we characterized a type I PME, PME34, which plays an important role in controlling stomatal aperture to regulate the transpiration rate during the heat response (Huang et al., 2017). In this study, we revealed a guard cell-specific type II PME, PME53, required for stomatal density, movement, and heat response control. Transcript accumulation of *PME53* is induced by exogenous ABA treatment, and the *PME53* promoter mediates strong induction of GUS reporter expression in guard cells. Using a *trans*-activation assay, we showed that the expression level of *PME53* is significantly affected by SCRM and MUTE, which function as master regulators of stomatal development. This work highlighted that PME53 is involved in fine-tuning pectin methylesterification, modulating stomatal cell fate and patterning, and aiding in environmental adaption.

## Materials and Methods

### Plant Materials and Growth Conditions

Arabidopsis (*Arabidopsis thaliana*) wild type (Col-0) and homozygous T-DNA insertion mutants *PME53* (SALK_150305) and *SCRM* (SALK_003115) were obtained from the Biological Resource Center (ABRC). Sterile seeds were placed on solid half-strength Murashige and Skoog basal medium (1/2 MS; Sigma M5519) (Murashige and Skoog, 1962) containing 1% sucrose and 0.8% phytagel (Sigma). Plants were grown in growth chambers at 22 to 24°C under an 8-h dark/16-h light cycle at an intensity of 80 to 100 μmol m^2^ s^–1^. Transgenic plants of *PME53*-promoter*:GUS* with pCAMBIA1391Z vector in Col background and CaMV *35S:PME53-3xFLAG* overexpression plants with pCAMBIA3300 vector (Karimi et al., 2002) in a *pme53* mutant background were generated by *Agrobacterium tumefaciens* GV3101-mediated transformation and by the floral dip method (Clough and Bent, 1998).

### RNA Preparation, cDNA Synthesis, and Quantitative Real-Time PCR

Seven-day-old seedlings were ground with liquid nitrogen and suspended with TRIZOL reagent (Invitrogen) for RNA preparation and removed the contaminating DNA involved with TURBO DNA-free kit (Applied Biosystems). cDNA synthesis was performed with the high-capacity cDNA reverse transcription kit (Applied Biosystems). PCR primers were designed using the Primer3^2^. Quantitative real-time PCR (q-PCR) reactions were analyzed by the 7500 Fast Real-Time PCR System (Applied Biosystems) with the q-PCR mix of SYBR Green Supermix (BIO-RAD). The internal control for normalization was *PP2AA3* (*PP2A*; At1g13320) (Czechowski et al., 2005).

### Constructs of Transactivation Assay and amiRNA-Mediated Gene Suppression

A 1.974-kb promoter region of *PME53* was amplified and cloned into the pGreenII 0800-LUC vector through *Bam*HI and *Hin*dIII sites for *PME53*-promoter*:LUC* (Firefly luciferase) fusion then used for transactivation assay. The automated design of artificial miRNAs (amiRNA) was according to the WMD3 (Web MicroRNA Designer)^3^. The 21mer amiRNA of the tester was cloned into the pRS300 vector which contains the miR319a precursor (Schwab et al., 2006). Sequence-verified amiRNA was cloned into the Gateway vector pCR8™/GW/TOPO and was recombinant into a destination vector pCAMBIA3300-CHF-DEST which was regulated by the 35S promoter. The amiRNA-containing vector was used for suppression of tester gene in transactivation assay (Karimi et al., 2002).

### Pectin Methylesterase Activity Assay

Fifty of 7-day-old seedlings were ground and suspended with 100 μL extraction buffer (1 M NaCl, 0.1 M citric acid, and 0.2 M Na_2_HPO_4_, pH 5.0) for 5 min and centrifuged at 13,800 × *g* for 15 min. The supernatant was collected and cell wall protein concentrations were determined according to the method of Bradford (1976). The enzymatic PME activity was quantified by a gel diffusion assay as described (Downie et al., 1998; Bethke et al., 2014; Lionetti, 2015) with some modification. 10 μL of 3.75 μg protein extract was loaded into the 0.3 mm well on the 20 mL gel prepared in McIlvaine buffer adjusted to pH levels of 4 to 8, which contains 2% agarose and 0.1% of high methylesterified pectin (≥85% esterified pectin from citrus fruit; P9561, Sigma-Aldrich). After 16 h incubation at 28°C, the gels were stained with 10 mL of 0.05% (w/v) ruthenium red (Sigma-Aldrich) for 1 h and de-stained with distilled water. The calibration curve of PME activity was established with a detection range from 2 to 8 μg/mL of cell-wall proteins. The PME activity was calculated by measuring the stained area (cm^2^) by ImageJ software^4^.

### Stomatal Aperture Measurement

The stomata tape-peel method was used for guard cell sample preparation as described (Lawrence et al., 2018). Fully expanded leaves of 4-week-old soil-grown plants were used per line for each treatment. Briefly, mature leaves (7th and 8th) were selected and attached to two pieces of tapes to both sides, then gently peeled apart the two pieces of tapes. The peels were incubated in the stomata opening buffer (50 mM KCl, 10 mM MES-KOH, pH 6.2) at 22°C for 2.5 h with light for equilibration and stomata opening. Afterward, the peels were further treated with 10 μM ABA for the appropriate treatment time. After treatment, the stomata aperture was observed by a light microscope and measured by ImageJ software^4^. The data were presented in comparison to the corresponding controls.

### Determination of Water Loss Assay

Water loss and standardized water contents in the full opened leaf by following the procedure described previously (Fujita et al., 2005). Briefly, 40 detached 7th and 8th rosette leaves from 4-week-old soil-grown seedlings were kept in a petri dish. The transpiration (water loss) measurement was standardized (%) and then calculated as [(FWi-DW)/(FWo-DW)] × 100, where FWi and FWo are fresh weight for any given interval and original fresh weight, respectively, and DW is dry weight. These tests were conducted on the laboratory bench at 24 to 26°C and 60 to 70% relative humidity.

### Histochemical Glucuronidase Staining

A 1.974-kb promoter region of *PME53* was fused with reporter β-glucuronidase (GUS) gene in pCAMBIA1391Z (CAMBIA) and used for the tissue-specific expression analysis, and the GUS staining method was performed as described previously (Weigel and Glazebrook, 2002). The provided images of GUS-stained tissues represent the typical results of at least six independent transgenic lines.

### Protoplast Isolation, Transient Expression and Transactivation Assay

Protoplast isolation and transactivation assay were carried out as previously described (Yoo et al., 2007). For the transactivation assay, 4 × 10^4^ protoplasts were transfected with 5 μg plasmid DNA or 10 μg amiRNA-containing plasmid in a total 20 μL reaction were conducted. Transfected protoplasts were incubated at 22 to 24°C under a light condition at 80 to 100 μmol m^–2^ s^–1^ for 14 to 16 h. The YFP signal was detected by Zeiss LSM780 confocal microscope. The dual-luciferase activity was measured by quantifying LUC and REN (Renilla luciferase) activities with the microplate reader Infinite 200 PRO (TECAN), according to the manufacturer’s instructions (Promega). The *35S* promoter-driven *REN* gene in the pGreen0800-LUC vector is used as an internal control.

### Subcellular Localization in Onion Epidermal Cells and Agroinfiltration-Based Transient Gene Expression in Tobacco

Transgene *35S:PME53-YFP* with pEarleyGate101 vector was transformed into onion epidermal cells by particle bombardment as described previously (Guan et al., 2004; Huang et al., 2017). Approximately 2.5 μg of DNA was coated onto 0.6 μm gold particles (Bio-Rad) and transiently introduced into onion epidermal cells with a helium biolistic particle-delivery system (PDS-1000; Bio-Rad). The bombarded cells were kept at 26°C in the dark for 16 h. The plasmolysis of onion epidermal cells was induced by addition of 0.8 M mannitol solution for 30 min. 4-week-old tobacco (*Nicotiana benthamiana*) plants were infiltrated 100 μL recombinant Agrobacteria per spot by needless syringe then keep plants at 22 to 24°C with a 16 h/8 h light/dark photoperiod for 2 to 3 days (Sparkes et al., 2006). The signals were then observed and photographed by Zeiss LSM780 confocal microscope.

### Heat Treatments

Thermotolerance assays of seven-day-old seedlings were performed as previously described (Charng et al., 2006; Hsu et al., 2010; Huang et al., 2016). For the acquired thermotolerance test, plates (20-mL 1/2 MS) were preheated at a 37°C sub-lethal heat shock for 1 h and allowed to recover at 22°C for 2 h before a 44°C lethal heat shock for 170 to 180 min. Healthy growing seedlings were counted 10 to 14 days after the heat treatment.

### Statistical Analysis

All experiments were repeated independently at least three times. Statistical analysis involved Student’s *t*-test and ANOVA with Tukey’s HSD *post hoc* test. *P* < 0.05 was considered statistically significant.

### Primers and Accession Numbers

Primers and accession numbers are given in Supplementary Table 1.

## Results

### Expression of *PME53* in Response to Abiotic Stress and in Developing Tissues

In the Arabidopsis microarray database, the expression profile of the predicted *PME* genes varied by ∼75% in response to abiotic and biotic stresses (Pelloux et al., 2007). Here, the guard-cell-specific microarray analyses side by side with a mesophyll-cell-specific microarray (Yang et al., 2008), 59 *PME* genes were assayed under ABA treatment. We showed that the expression of *PME53* increased significantly after treatment with 100 μM ABA for 1 h in guard cells, but not in mesophyll cells (Supplementary Figure 1). The Arabidopsis *PME34* gene, an ABA-regulated stomatal gene, was used as reference (Huang et al., 2017).

**FIGURE 1:**
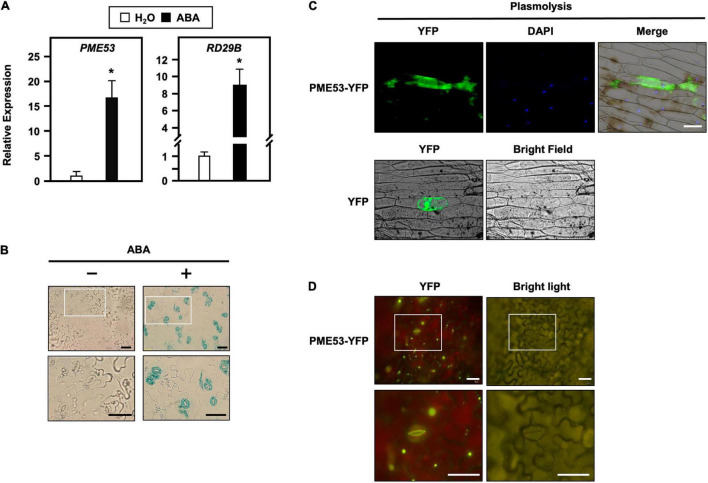
Transcriptional level, tissue-specific expression and subcellular localization of PME53. **(A)** 7-day-old seedlings were treated without (H_2_O control) or with 100 μM ABA for 1 h. The expression levels of *PME53* were analyzed by q-PCR. *RD29B*, an ABA-responsive gene, was used as reference. The fold change expression was normalized relative to level of the H_2_O control. Data are mean ± SE of three biological replicates. *Significant at *P* < 0.05 compared with the H_2_O treatment. **(B)** Cotyledons of the same *PME53*-promoter*:GUS* transgenic plants treated without (-) or with (+) 30 μM ABA for 3 h, and then specimens were immersed together in a staining solution. The magnification of the insert (white frame) is shown. Bars = 40 μm. **(C)** The onion epidermal cells were treated with mannitol for the plasmolysis assay. Subcellular localization analyzes of PME53-YFP by confocal microscopy. YFP was used as a control. Blue shows nuclei stained with 4′,6-diamino-phenylindole (DAPI). Bar = 200 μm. **(D)** Tobacco cells were infiltrated with Agrobacterium harboring *35S:PME53-YFP* transgene. The magnification of the insert (white frame) shows that PME53 is mainly localized in the inner wall layer of guard cells. Bars = 40 μm.

Seven-day-old Arabidopsis seedlings subjected to ABA, salt, and osmotic treatments were also conducted, and *PME53* expression levels were analyzed by q-PCR (Figure 1A and Supplementary Figure 2). After treatment with 100 μM ABA for 1 h, the expression levels of *PME53* increased 15-fold compared with the untreated plants (Figure 1A). In addition, treatment with 150 mM NaCl and 300 mM mannitol for 6 h resulted in significantly downregulated *PME53* expression (Supplementary Figure 2). Arabidopsis *RAD29B*, an ABA, salt, and osmotic-responsive gene, was used as reference.

The potential *cis*-elements in the 2-kb promoter region of *PME53* show multiple potential ABA response elements (ABREs), with the Plant Promoter Analysis Navigator (PlantPAN3)^5^ (Chow et al., 2019; Supplementary Figure 3). Therefore, the tissue-specific expression of *PME53* was analyzed in transgenic plants harboring a 1.974-kb promoter region of *PME53* fused with a reporter *GUS* gene. The *PME53*-promoter*:GUS* transgenic lines indicated that *PEM53* was ubiquitously expressed in the analyzed tissues (Supplementary Figure 4).

Notably, it was strongly upregulated after 3 h of treatment with 30 μM ABA in guard cells (Figure 1B). Together these results imply that *PME53* may play a role in the regulation of ABA-mediated stomatal responses.

### PME53 Resides in Apoplast and Inner Wall Layer of Guard Cell

Arabidopsis PME53 protein (UniProt: Q8VYZ3) belongs to a type-II PME that is predicted to have a signal peptide (SP; amino acids 1 to 28) along with the conserved PME domain (amino acids 29 to 383) according to the UniPort database^6^ (Supplementary Figure 5). Using the CaMV *35S:PME53-YFP* transgene, the onion epidermal cells were submitted to 0.8 M mannitol for 30 min to induce plasmolysis, indicating that the PME53 reporter was located in the apoplast (Figure 1C). Tobacco cells were infiltrated with *Agrobacterium* cells expressing the *35S:PME53-YFP* transgene, demonstrating that PME53 is primarily found in the inner-wall layer of guard cells (Figure 1D). These findings suggest that PME53 is a guard cell wall-localized protein that may aid in the opening and closing of stomatal pores in guard cells.

### Characterization of *PME53* T-DNA Insertion and *PME53*-Overexpression Lines

**FIGURE 2:**
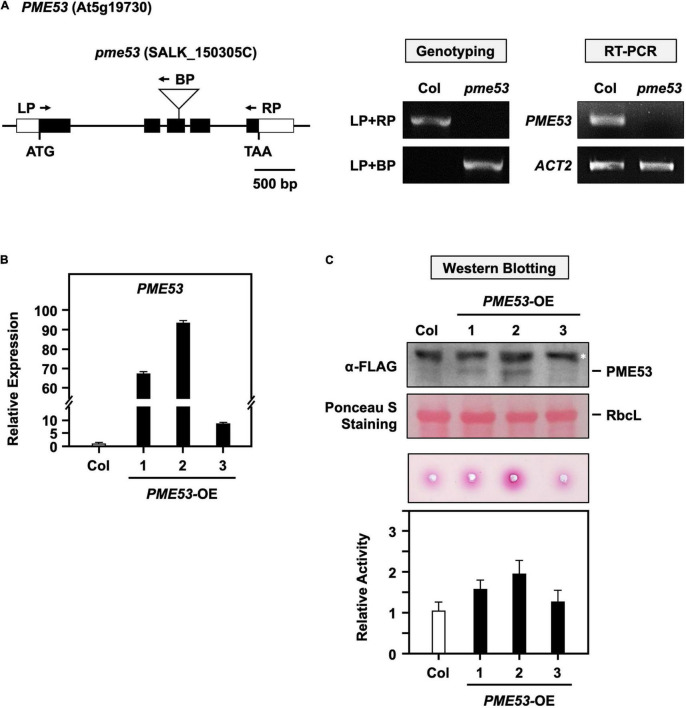
Characterization of *PME53* T-DNA insertion and its overexpression lines. **(A)** Schematic map of *PME53* gene and T-DNA insertion site. The white box, black box, and solid line indicate the UTR region, exon, and intron, respectively. ATG are shown as initiation codons. T-DNA insertion site is indicated as triangle, and primers for genotyping are indicated as arrows. RT-PCR involved the *PME53* gene-specific primers. *Actin2* (*ACT2*) was used as an input control. **(B)** The expression levels of *PME53* in three independent lines, *PME53*-OE1 to OE3, were analyzed by q-PCR. The fold change expression was normalized relative to level of the Col. **(C)** 7-day-old seedlings were used for PME53 protein level (top) and activity (bottom) analysis. Immunoblot stained with Ponceau S and the ribulose bisphosphate carboxylase large subunit (RbcL) was as a loading control. Western blotting analysis using anti-FLAG antibody. *Indicates non-specific immune signals. The fold change PME activity was normalized relative to level of the Col.

Thus, the role of PME53 physiological has mainly been studied through its T-DNA insertion and overexpression lines (Figure 2). One *PME53* homozygous T-DNA insertion mutant was screened by PCR-based genotyping and the null-mutant lines were confirmed by RT-PCR (Figure 2A). *PME53* fused with a 3xFLAG tag to the C-terminus and driven by a *35S* promoter to generate the *PME53-*overexpression (OE) lines in a *pme53* mutant background (*PME53-*OE). Three independent OE lines, *PME53-*OE1 to OE3, were obtained and characterized by q-PCR (Figure 2B) and western blotting with an α-FLAG antibody (Figure 2C, top). These three *PME53-*OE lines showed an increased PME activates compared with the wild-type (Col) plants (Figure 2C, bottom), then the *PME53-*OE2 plants used for the following experiments.

### PME53 Is a Functional Pectin Methylesterase With an Optimal Activity at Acidic pH Value

The evidence indicates that apoplastic pH is an important determinant of PME activity and contributes to the de-methylesterification of cell wall pectins (Bosch and Hepler, 2005). The PME activity was measured by a gel diffusion assay (Supplementary Figure 6, top), and the calibration curve of PME activity was linear (*R*^2^ = 0.9831) over the range from 2 to 8 μg/mL of cell wall proteins (Supplementary Figure 6, bottom). A 3.75 μg protein extract used for the following PME activity assay.

**FIGURE 3:**
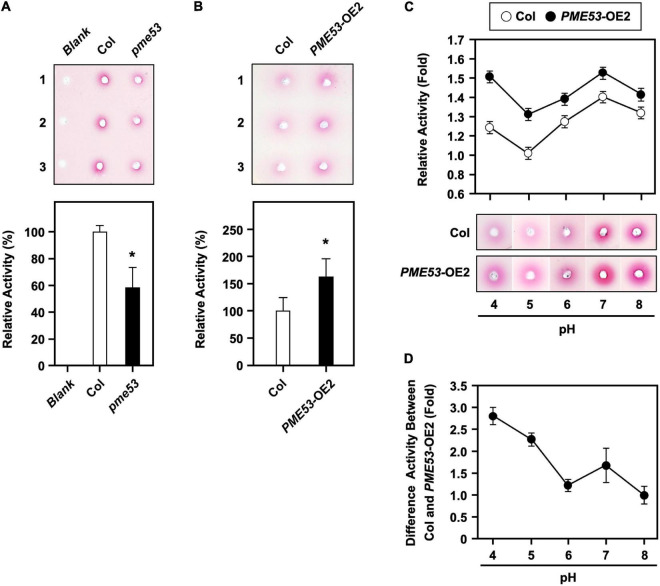
PME activity assay in *pme53* mutant and *PME53*-overexpression plants. PME activity was analyzed **(A)** in Col and *pme53* and **(B)** in Col and *PME53-*OE2 plants. The ruthenium red-stained zone diameters resulting from the hydrolysis of esterified pectin in an agarose gel by diffusion were photographed and measured. The fold change PME activity was normalized relative to level of the Col. Data are mean ± SE of three biological replicates. *Significant at *P* < 0.05 compared with the Col. Blank, the buffer used only. **(C)** PME activity analyzed at pH levels of 4 to 8. PME activity was normalized to the weakest activity at pH 5 of the Col. **(D)** The optimal PME53 activity at different pH levels was summarized. Data are mean ± SE of three biological replicates.

Here, we demonstrated that *pme53* showed a significant reduction in PME activity as compared with the Col (Figure 3A) but significantly increased PME activity in *PME53*-OE2 plants (Figure 3B). The optimal PME activity at different pH levels in the Col and *PME53-OE2* plants was analyzed (Figure 3C). PME53 activity was highest at acidic pH levels of 4 to 5 and decreased with alkaline pH of 8 (Figure 3D). This result indicated that PME53 protein is a functional PME with optimal PME activity at a more acidic pH value.

### Phenotyping of *pme53* Mutant Plants

**FIGURE 4:**
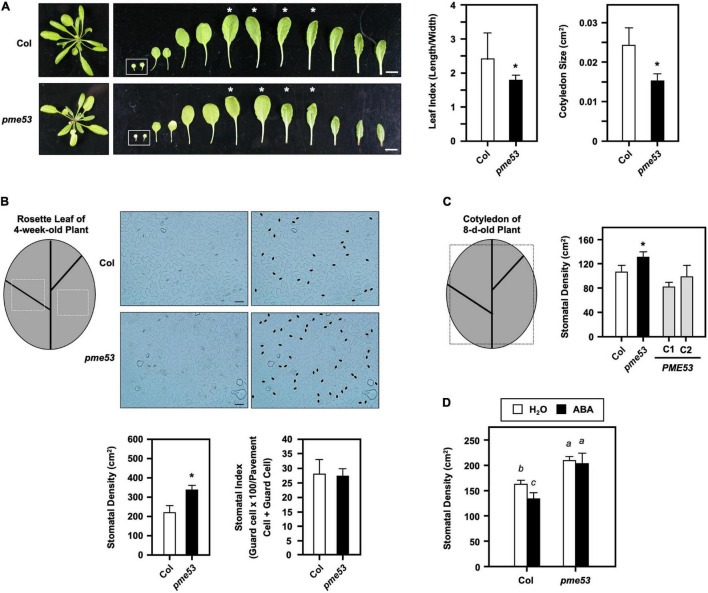
Phenotyping of *pme53* mutant plants. **(A)** 4-week-old plants and leaves were photographed. Cotyledons are shown in the inset. Leaf index (length/width) was determined by the ratio of the length to width of the 3rd and 4th pair of rosette leaves (asterisk indicated) and the size of the 7-day-old cotyledon (cm^2^) was measured. *n* = 50 cotyledons. Bars = 1 cm. **(B)** Epidermal cells on the abaxial surface of 3rd and 4th pair leaves of 4-week-old plants were photographed. Stomata were highlighted in black ovals. Bars = 50 μm. Stomatal density (stomata/cm^2^) and stomatal index (guard cell × 100/pavement cell + guard cell) were measured. **(C)** Stomatal density in the abaxial surface of the cotyledon of 8-day-old plants were measured. *PME53*-C1 and -C2 are *PME53*-complemented lines generated in *pme53* background. The frames are indicated that the regions used for the stomatal density measurement. *Significant at *P* < 0.05 compared with the Col. *n* > 10 leaves and cotyledons. **(D)** Seedlings were grown together then applied with H_2_O (control) or 2.5 mL 10 μM ABA, at day 4 after planting. Stomatal development (stomatal/cm^2^) in cotyledons was analyzed on day 7. Data are mean ± SE of three biological replicates. *n* > 10 cotyledons. Statistical significances among groups are indicated using different letters (Tukey’s HSD test).

A comparison of the rosette leaf index showed that the leaf width to length ratio in the leaves of *pme53* was significantly smaller than that of the Col (Figure 4A). In particular, the size of the cotyledon (cm^2^) of 7-day-old *pme53* appeared smaller and shorter than that of the Col.

The GUS staining results showed that the *PME53* was highly expressed in the vascular tissue of cotyledons (Supplementary Figure 4), and therefore, the vascular network complexity of cotyledons was analyzed (Supplementary Figure 7). According to previous studies, the number of closed areoles (2, 3, or 4) formed by secondary veins and the number of vein branches/incomplete areoles in the proximal (closest to the petiole) part of the cotyledon (Supplementary Figure 7, top) were used to classify vein complexity patterns in cotyledons (Roschzttardtz et al., 2014). In comparison to the Col, *pme53* plants had more areoles of 4.1, 4.0, and 3.1, but fewer areoles of 2.2, 3.0, and 2.1 (Supplementary Figure 7, bottom). As a result, *pme53* seedlings with 7-day-old cotyledons had abnormal vein networks. These data imply that PME53 may determine the continuity and pattern formation of vascular tissues in Arabidopsis.

### Stomatal Density in *pme53* Mutant Plants

The stomatal density (number of stomata/cm^2^), stomatal index (guard cell × 100/pavement cell + guard cell), and pavement cell size were analyzed on the leaf epidermal layers of *pme53* (Figure 4B). The stomatal number and pavement cell size were largely affected and were analyzed on the 7th and 8th leaves of 4-week-old *pme53* compared with the Col (Figure 4B). Higher stomatal density was found in *pme53*; however, the stomatal index was not significantly affected (Figure 4B). Notably, two complementation of *pme53* mutation lines, *PME53-*C1 and C2, restored the high stomatal density phenotype in *pme53* to that of the Col (Figure 4C).

It is known that ABA treatment reduces the number of stomata cells in Col plants (Tanaka et al., 2013). Four-day-old *pme53* plants were treated with H_2_O (control) or 10 μM ABA for 3 days, and then the stomatal density of cotyledon was measured. The effect of ABA on reduced stomatal density was not found in *pme53* compared with that in Col plants (Figure 4D).

### Stomatal Size and Movements Control in *pme53* Mutant Plants

**FIGURE 5:**
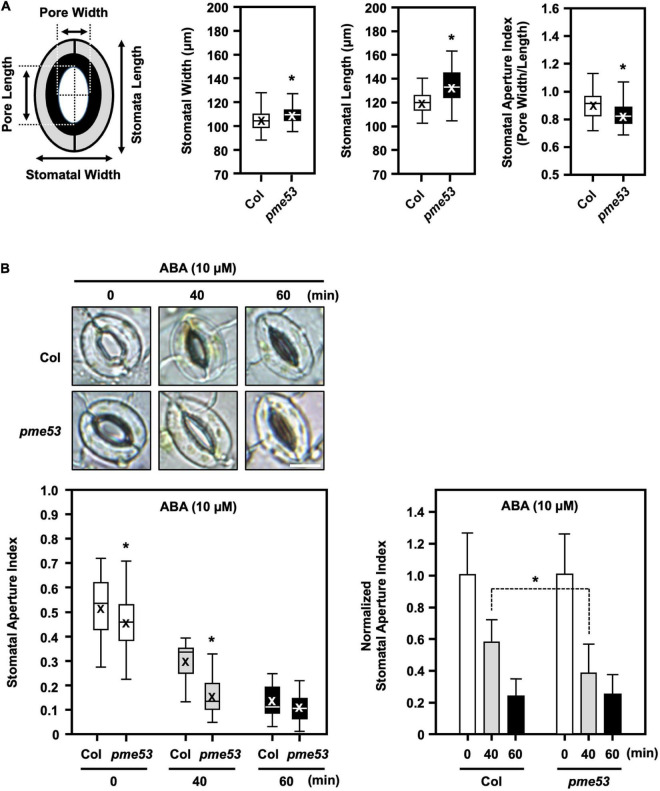
Stomata maximal-opening ability and movement in *pme53* mutant plants in response to ABA. Schematic representation of stomata pore measurement. **(A)** Epidermis peels of the fully expended 4-week-old plants incubated in an opening buffer under light for 3 h then the stomata maximal-opening ability was analyzed stomatal length (μm) and width (μm), as well as the stomatal aperture index (pore width/length), were measured. *n* > 80 stomata. **(B)** Stomatal movement in response to 10 μM ABA for 40 or 60 min was measured. The stomata aperture index and the normalized stomata aperture index were measured. *n* > 80 stomata. Representative images of stomata were shown. Bars = 10 μm. Data are mean ± SE of three biological replicates. *Significant at *P* < 0.05 compared with the Col (ANOVA Tukey-Kramer *post hoc* test).

Phenotyping of the stomata in *pme53* was conducted to measure the structural features, such as stomatal opening length and width (Figure 5). After incubation in the opening buffer, we showed that stomata lengths were longer and wider in *pme53* than in Col plants (Figure 5A). Notably, *pme53* showed statistically significant decreases in stomatal aperture compared with the Col (Figure 5A). However, the stomatal aperture in three *PME53*-OE lines was similar to that in *pme53* plants (Supplementary Figure 8).

In response to 10 μM ABA treatment, *pme53* showed more sensitivity to stomatal closure than Col plants (Figure 5B). Our results suggest that PME53 plays a predominant role in the regulation of stomatal patterning and movement control.

### *PME53* Controls in Stomata Development

**FIGURE 6:**
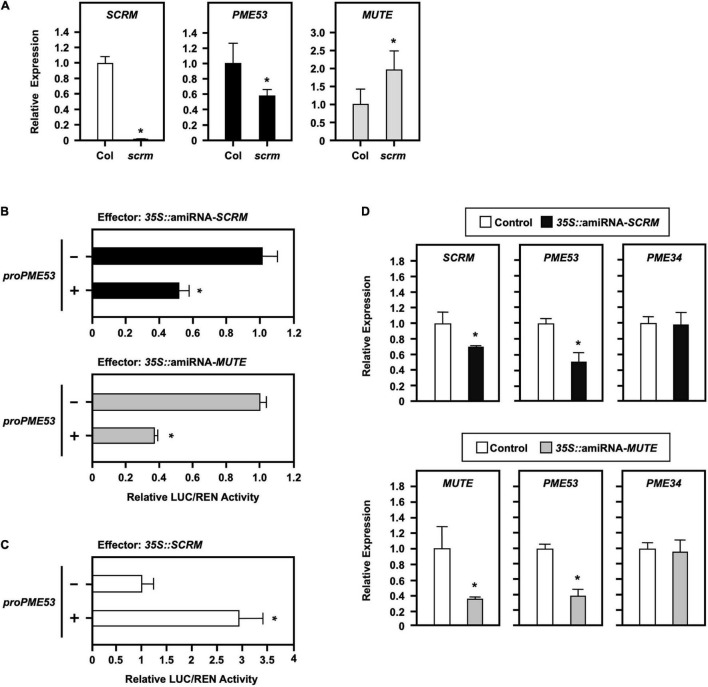
Effect of the stomatal-positive master regulator SCRM on *PME53* expression. **(A)** The expression levels of *SCRM*, *PME53*, and *MUTE* in 7-day-old seedlings of Col and *scrm* (the null-mutant line) were analyzed by q-PCR. The fold change expression was normalized relative to the level of Col. **(B,C)** The *35S:Effector* of artificial miRNA mediated the suppression of *SCRM* and *MUTE* expression (amiRNA-*SCRM* and amiRNA-*MUTE*) and *35S:SCRM*, were used for the *trans*-activation analysis of *PME53-*promoter*:LUC* reporter in Arabidopsis protoplasts, respectively. The fold change expression was normalized relative to the activity of LUC/REN using the dual-luciferase system. *Significant at *P* < 0.05 compared with the mock control (-). **(D)** The expression levels of *SCRM*, *MUTE*, *PME53*, and *PME34* were analyzed by q-PCR. *PME34* was used as a negative reference control. Data are mean ± SE of three biological replicates.

Abscisic acid-mediated regulation of stomatal density acts upstream to regulate the expression of *SCREAM* (*SCRM*, also known as *ICE1*) and *MUTE* genes, which are master regulators for stomatal formation in Arabidopsis. One *SCRM*-knockout mutant (*scrm*; *ice1-2*) was confirmed by q-PCR analysis (Figure 6A, left) and used to elucidate the requirement of the *PME53* gene expression. In the *scrm* mutant, *PME53* expression was reduced (Figure 6A, middle), whereas *MUTE* expression was increased (Figure 6A, right).

Furthermore, a 1.974-kb promoter region of *PME53* fused with a reporter Firefly luciferase (LUC) and co-transfected with the effector of a *35S*-driven artificial miRNA, amiRNA-*SCRM* and amiRNA-*MUTE*, to mediate the downregulation of *SCRM* and *MUTE* analyzed in Arabidopsis protoplasts (Figure 6B). Renilla luciferase (REN) activity was used as the input control, and the activity of LUC/REN normalized the fold of expression. The results of transactivation assays highlighted that the overexpression of amiRNA-*SCRM* and amiRNA-*MUTE* led to the downregulated expression of *PME53* (Figure 6B). In addition, overexpression of *SCRM* exerted positive effects on the expression of *PME53* (Figure 6C). The overexpression of amiRNA-*SCRM* and amiRNA-*MUTE* suppressed the expression of *SCRM* and *MUTE*, which led to the downregulation of *PME53* gene expression (Figure 6D). Arabidopsis *PME34*, a stomatal-density-independent gene, was used as a negative reference control (Huang et al., 2017).

### *pme53* Mutant Plants in Response to Heat Stress

**FIGURE 7:**
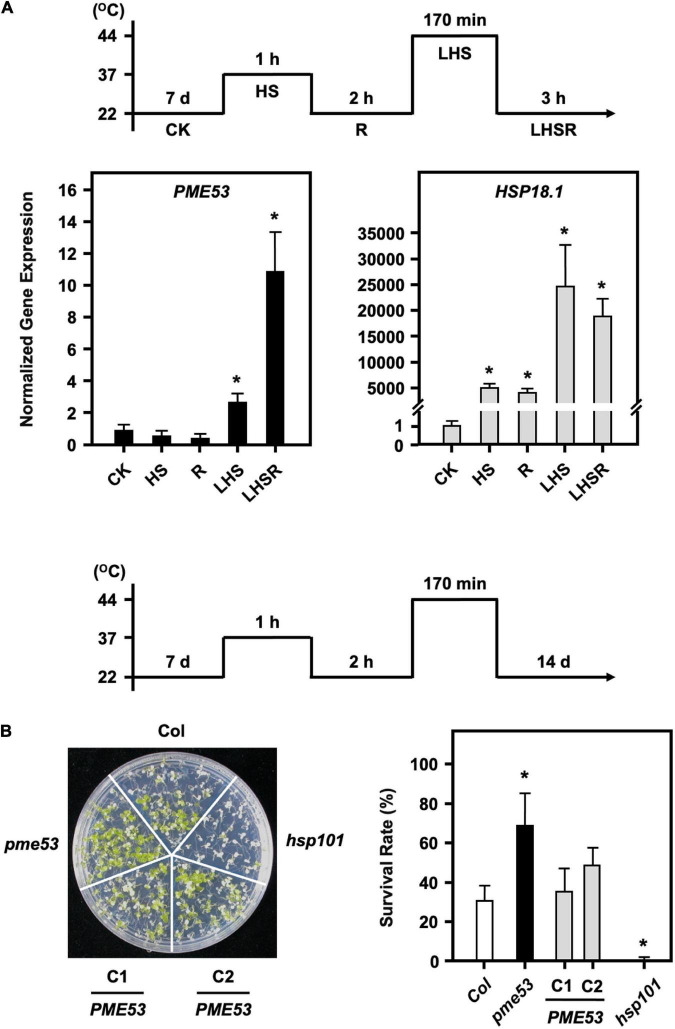
*pme53* mutant and its complementation lines in response to heat stress. The pictogram shows the heat stress regime. **(A)** The expression levels of *PME53* in 7-day-old seedlings were analyzed by q-PCR. *HSP18.1*, a heat-responsive gene, was used as reference. The fold change expression was normalized relative to the level of control (CK). Data are mean ± SE of three biological replicates. *Significant at *P* < 0.05 compared with the control. **(B)** Seedlings were photographed and survival was measured at 14 days after the LHS treatment. *PME53*-C1 and -C2 are *PME53*-complemented lines generated in *pme53* background. *hsp101*, a heat-sensitive mutant, was used as reference. Data are mean ± SE of three biological replicates (*n* = 50 seedlings). *Significant at *P* < 0.05 compared with the Col.

Stomata regulate gas exchange by allowing water vapor to leave the plant and CO_2_ to enter to keep the leaf temperature stable. Although the *pme53* had a higher stomatal density and decreased in stomatal aperture, preliminary data indicated that *pme53* did not significantly influence water transpiration rates during 2 h recovery from a 1-h 37°C mild heat shock treatment (Supplementary Figure 9). It has been reported that *PME34* is required for proper heat response due to its role in promoting stomatal movements, as we previously reported (Huang et al., 2017). To gain insight into whether PME53 plays a role in the heat response, we showed that the expression of *PME53* gene was slightly downregulated on 1-h 37°C heat shock treatment (Figure 7A). However, *PME53* expression was significantly increased after a 44°C-lethal heat shock (LHS) and 3-h recovery from the LHS (LHSR) (Figure 7A). *HSP18.1*, an HS-responsive marker gene, was used as reference. Notably, the *pme53* exhibited a less heat-sensitive phenotype with a higher survival rate compared with the Col (Figure 7B), while complementation lines, *PME53-*C1 and C2, restored the defective heat-responsive phenotype similar to that of Col plants. *hsp101*, a heat-sensitive mutant, was used as reference.

We showed that *pme53* was properly perceived and responsive to the heat shock at 37°C for 1 h. In agreement with the expression levels of major heat-responsive genes, including *HSP101*, *HSP90*, *HSP70*, and *HSP18.1*, were unaffected compared with the Col (Supplementary Figure 10).

### Status of De-Methylesterified Pectins in *pme53* Mutant Plants in Response to Heat Stress

To assess the correlation between patterns of pectic enzyme production and heat response, ruthenium red (RR), a cationic reagent, has been used to visualize polysaccharides on the plant cell wall (Wu et al., 2010; Suzuki et al., 2011). Firstly, we characterized pectic substances in the cotyledon of 7-day-old seedlings by RR staining *in situ*, *pme53* showed a less RR stain-abled demethylesterified pectin than the Col after a 44°C LHS and 3-h recovery from the LHS (LHSR) (Supplementary Figure 11A).

**FIGURE 8:**
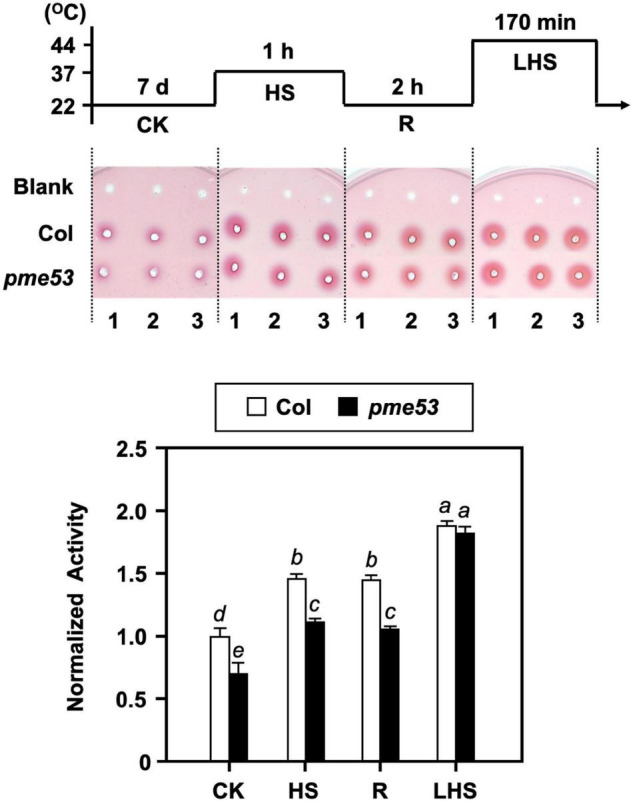
PME activity in *pme53* mutant plants in response to heat stress. The pictogram shows the heat stress regime. PME activity in 7-day-old seedlings was analyzed. The ruthenium red-stained zone diameters resulting from the hydrolysis of esterified pectin in an agarose gel by diffusion were photographed and measured. The fold change PME activity was normalized relative to level of the Col control (CK). Blank, the buffer used only. Data are mean ± SE of three biological replicates. Statistical significances among groups are indicated using different letters (Tukey’s HSD test).

Our previous studies confirmed that the inward movement of Ca^2+^ and the recovery of heat shock-triggered released apoplastic Ca^2+^ accompanied by PME activity are required for thermotolerance (Wu et al., 2010). Thus, the total PME activity in *pme53* was analyzed under heat shock (Figure 8). The PME activities in Col were induced in response to heat shock treatments at 37°C and 44°C, while that of *pme53* was significantly reduced under normal growth conditions, response to 37°C heat shock, and recovery form 37°C heat shock compared with the Col (Figure 8, HS and R). Notably, the total PME activity was activated in *pme53* during 44°C-LHS treatment similar to the Col (Figure 8, LHS), we suggest that other unclarified PMEs were activated to compensate for the PME53 activity in *pme53*. Also, the *PME53*-OE2 has been shown a similar PME activity pattern as that of Col plants in response to heat stress (Supplementary Figure 11B).

### PME53 May Be Involved in Pectin Methylesterase-Mediated Inward Movement of Ca^2+^

**FIGURE 9:**
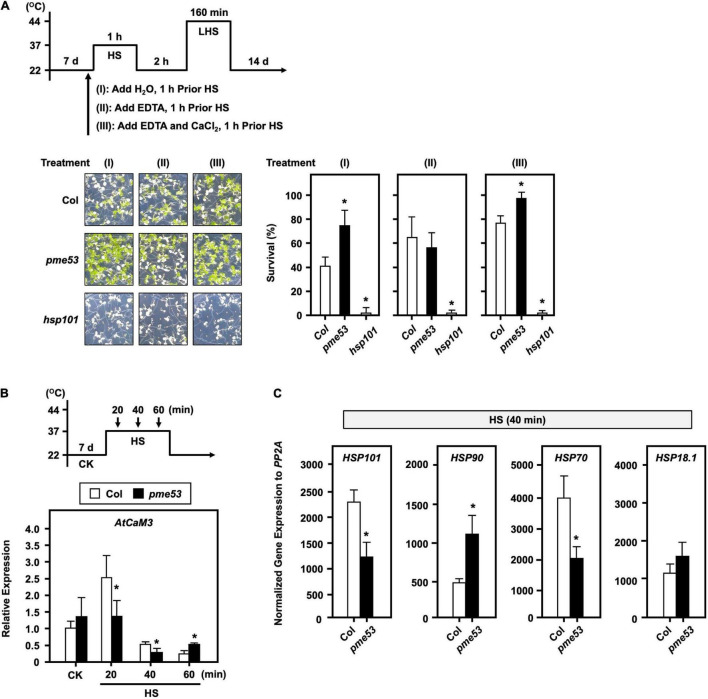
The effect of EDTA and Ca^2+^ on the phenotype of *pme53* plants and expression levels of *AtCaM3* in response to heat stress. The pictogram shows the heat stress regime. **(A)** Seedlings were applied with 2.5 mL H_2_O, 20 μM EDTA, or 20 μM EDTA with 15 mM CaCl_2_ solution, 1 h before HS treatment. Seedlings were photographed and survival was measured at 14 days after 44°C-LHS treatment. *hsp101*, a heat-sensitive mutant, was used as reference. Data are mean ± SE of three biological replicates (*n* = 50 seedlings). *Significant at *P* < 0.05 compared with the Col. **(B)** 7-day-old seedlings in response to 37°C HS for 20 to 60 min. The expression levels of *AtCaM3* were analyzed by q-PCR. The fold change expression was normalized relative to the level of Col control (CK). **(C)** The expression levels of heat-responsive genes after 40-min 37°C HS were analyzed by q-PCR. Data are mean ± SE of three biological replicates. *Significant at *P* < 0.05 compared with the Col.

To confirm that the thermotolerant phenotype of *pme53* is related to the PME-mediated inward movement of Ca^2+^. 7-day-old seedlings were treated with 20 μM EDTA or 20 μM EDTA + 15 mM CaCl_2_ before heat stress (Figure 9A). Our results demonstrated that the EDTA-treated seedlings in *pme53* showed a thermoresponsive phenotype similar to that of Col plants. Meanwhile, treatment with Ca^2+^ to antagonize the effect of EDTA restored the less heat-sensitivity phenotype of *pme53* that was similar to H_2_O control treatment.

In addition, Arabidopsis calmodulin AtCaM3 is a key member of the Ca^2+^-mediated heat stress signaling pathway (Zhang et al., 2009). The Ca^2+^-induced *AtCaM3* gene expression reached its maximum level after 20 min 37°C heat stress in Col; however, it significantly reduced expression in *pme53*, which was much lower than that of Col (Figure 9B). The expression level of *HSP90* was increased, while *HSP70* and *HSP101* were decreased in *pme53* significantly than that of Col at the early 40 min 37°C heat stress (Figure 9C), indicating that *PME53* may coordinate with heat stress signaling, which is involved in Ca^2+^-pectate reconstitution to render thermotolerance in plants.

### Abscisic Acid Pre-treated *pme*53 Mutant Plants in Response to Heat Stress

**FIGURE 10:**
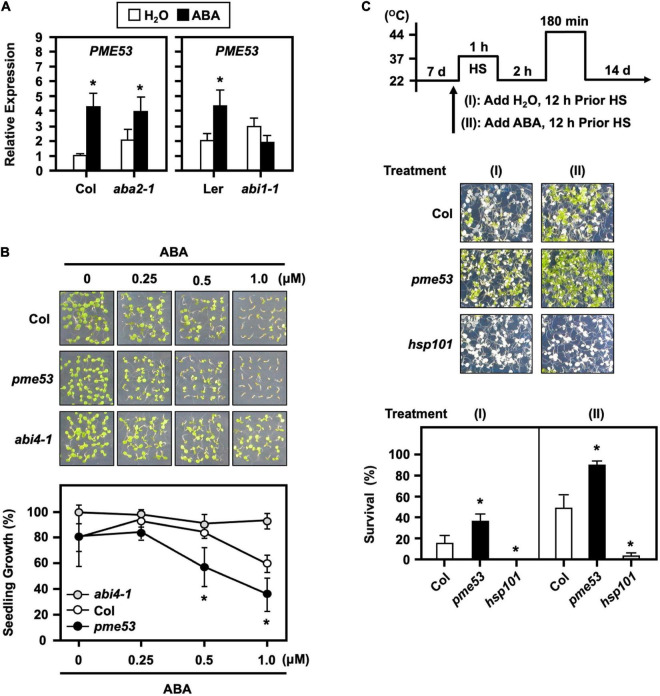
Transcriptional levels of *PME53* in ABA-deficient (*aba2-1*) and ABA-insensitive (*abi1-1*) mutant plants and the effect of ABA on *pme53* mutant post-germination growth and in response to heat stress. **(A)** 7-day-old seedlings were treated without (H_2_O control) or with 30 μM ABA for 3 h. The expression levels of *PME53* were analyzed by q-PCR. The fold change expression was normalized relative to their corresponding wild-type (Col or Ler) plants. Data are mean ± SE of three biological replicates. *Significant at *P* < 0.05 compared with the Col or Ler. **(B)** Seedling growth without or with 0.25 to 1 μM ABA on plates was photographed and measured at day 7 after planting. *abi4-1*, an ABA-insensitive mutant, was used as reference. **(C)** The pictogram shows the heat stress regime. Plates were incubated with 2.5 mL H_2_O or 10 μM ABA solution, 12 h before HS treatment. Seedlings were photographed and survival was measured at 14 days after the LHS treatment. *hsp101*, a heat-sensitive mutant, was used as reference. Data are mean ± SE of three biological replicates (*n* = 50 seedlings). *Significant at *P* < 0.05 compared with the Col.

To verify that ABA signaling is required for the expression of *PME53*, we examined ABA-deficient (*aba2-1*) mutant and ABA-insensitive (*abi1-1*) mutant plants by ABA treatment. The expression level of *PME53* was significantly affected in *abi1-1* (Figure 10A). We also showed that the *pme53* were more sensitive to 0.25 and 1.0 μM ABA treatments, with lower post-germination growth rates than the Col (Figure 10B). These results indicate that ABA perception and response are required for the appropriate expression of *PME53*. Arabidopsis ABA-insensitive 4 mutant (*abi4-1*) was used as a reference control (Söderman et al., 2000).

The effects of pre-treatment with ABA on plants responses to heat have been investigated, and it has been shown that plants have a better survival rate than untreated (Huang et al., 2016). Here, a 10 μM ABA applied 12 h before the heat stress conferred an increased thermotolerance in the Col plants (Figure 10C). Notably, the ABA-pretreated *pme53* showed a higher survival rate than that of the untreated plants. The results implied that the *PME53* gene was necessary and not functionally redundant in the ABA-mediated heat response.

## Discussion

Cell wall enzymatic modification of the pectin network in the functional properties of guard cell walls has an important effect on normal stomatal function. Guard cell walls in *pme6* plants are enriched in methylesterified pectin and decrease the response to trigger stomatal movement (Amsbury et al., 2016). This indicates that the mechanical change in the guard cell wall requires de-methylesterification of pectin, indicating the significant role of PMEs in stomatal function. In the current study, we identified a *PME* gene, *PME53*, which is an ABA-responsive gene highly expressed in guard cells, and plays a significant role in stomatal development through the regulation of the core stomatal SCRM and MUTE transcription factors, and modulating the flexibility of the guard cell wall for elevated temperature adaptation. Hence, we determined that the function of PME53 showed the significance of ABA-mediated changes in guard cell walls involving the inflation characteristics of guard cell walls. Thus, the integration of ABA and PME activity is important for controlling stomatal functions by controlling guard cell wall plasticity in Arabidopsis.

### *PME53*, a Guard-Cell-Specific Gene, Is Involved ABA-Induced Stomatal Aperture

The molecular mechanisms underlying ABA-mediated stomatal aperture, thereby inducing adaptation to stress conditions, have been extensively investigated (Wasilewska et al., 2008; Hubbard et al., 2010; Lee and Luan, 2012). In addition, signaling by stress phytochrome ABA is involved in the activation of heat-responsive genes and acquired thermotolerance (Baron et al., 2012; Huang et al., 2016; Zandalinas et al., 2016). It has been shown that ABA changes heat-induced stomatal opening toward a higher threshold temperature, and this opening at elevated temperatures was fully reversible. It can be concluded that elevated temperatures can stimulate ABA-mediated stomatal opening, which has an opposite role to ABA in promoting stomatal closure under drought stress (Feller, 2006; Reynolds-Henne et al., 2010). Multiple ABRE domains were found in the promoter of *PME53* (Supplementary Figure 3), which was highly induced by exogenous ABA treatment (Figure 1A). *PME53* exhibited a tissue-specific pattern that was highly expressed in guard cells (Supplementary Figure 1 and Figure 1B), indicating that PME53 acts as a positive regulator of ABA signaling in guard cells.

The role of ABA in the regulation of guard cell wall structure, which determines stomatal aperture changes in response to stress, is still unknown. We found that the stomatal aperture in *pme53* was smaller than in Col plants (Figure 5A). Furthermore, in response to ABA treatment, the stomatal movement was more sensitive in *pme53* than in Col plants (Figure 5B), implying a strong and positive correlation between ABA-mediated stomatal aperture and PME53 function. ABA production and stomatal sensitivity to ABA were drastically reduced in *aba2-1* and *abi1-1* (Koornneef et al., 1982, 1984). Comparison of sensitivity to ABA inhibition of germination on *pme53* greatly increased ABA levels and significantly lowered the germination rate compared to Col and ABA-insensitive 4 (*abi4-1*) mutant plants (Figure 10B). As a result, *PME53* may contribute to the initiation of stomatal aperture control under unfavorable stress conditions as an ABA-inducible gene.

### *PME53* Involves in Stomatal Density Control and Stomatal Lineage Pathway

It is known that cell shape, cell size, and cell division of plant cell walls can be affected by intra- and extracellular cues; however, the relationship between pectin HGA methylesterification and epidermal cell growth remains elusive. The shape of pavement cells (non-stomatal cells) due to tension in the periclinal walls and their local reinforcement is linked to pectin de-methylesterification (Altartouri et al., 2019). The undulatory-shaped lobe of pavement cells can be affected by de-methylesterification of HGA with PME activity and interaction with Ca^2+^ in Arabidopsis epidermal cells (Haas et al., 2020). Epidermal pavement cells predominantly exhibit de-methylesterified pectin patterns, which can increase the stiffness of cell walls by crosslinking with Ca^2+^ (Bidhendi et al., 2019). Therefore, the formation of lobes in the epidermal pavement cells of leaves is associated with increased cell wall thickness, radial microfibril distribution, and HGA de-methylesterification (Majda et al., 2017). In Arabidopsis, un-methylesterified HGA is the predominant form of pectin in the guard cell wall, but highly methylesterified and Ca^2+^ cross-linked de-methylesterified HGA are present in the epidermal pavement and mesophyll cells, indicating that the mechanical properties of guard cells are largely dependent on the composition and structure of guard cell walls (Amsbury et al., 2016). Here, we showed that the leaf and pavement cells on the epidermis appeared to be different in *pme53* compared to Col (Figure 4A and Supplementary Figure 7). Our data indicated that PME53 may be involved in the morphogenesis of pavement cells by regulating the methylesterification status of pectin. In addition, the results of this study may provide crucial evidence underlying the initiation mechanism of cell shaping interrelated with the roles of de-methylesterified pectin of PMEs during the developmental process of the undulations of pavement cells.

Variation in stomatal density may arise due to genetic factors and/or growth to adapt to various environmental stresses (Bertolino et al., 2019). In addition, stomatal density directly affects stomatal efficiency; therefore, the development of stomata is tightly controlled by developmental and environmental cues to ensure precise stomatal lineage and patterning (Zhu et al., 2020). The stomatal apertures of *pme53* and *PME*-OEs plants were smaller than those of Col (Figure 5 and Supplementary Figure 8). The stomatal densities of *pme53* were significantly higher than those of Col plants (Figure 5B). An ABA-deficient *aba2-2* mutant had an increased stomata density within a smaller cotyledon, and reduced expansion of pavement cells, indicating that ABA negatively regulation in the initiation of stomatal development and ABA action on the enlargement of pavement cells in Arabidopsis cotyledons (Tanaka et al., 2013). The *pme53* exhibited an increased stomatal density and significantly reduced expansion of pavement cells. We suggest that PME53 may be involved in the ABA-mediated regulation of stomatal density and pavement cell expansion. Interestingly, the application of exogenous ABA did not reduce the stomatal density in *pme53* plants as that of the Col (Figure 4D), which is a different feature compared with the *aba2* mutant (Tanaka et al., 2013). Additionally, the expression levels of *PME53* were induced under ABA treatment in *aba2-1* but not *abi1-1* (Figure 10A), indicating that *PME53* plays a role in ABA perception and signaling. Besides, the *pme53* conferred a better survival rate than Col under heat stress (Figure 7B); therefore, we suggest that *pme53* exhibited a significantly higher stomatal density, correlating with remarkable resistance to temperature elevation.

Stomatal cell fate and patterning, which are controlled by key bHLH transcription factors, including SPCH, MUTE, FAMA, and their partners ICE1 and SCRM2, play a critical role in regulating sequential developmental decisions in Arabidopsis guard cell differentiation at different steps (Hofmann, 2008; Davies and Bergmann, 2014). Since SCRM form dimers with MUTE, FAMA, and SPCH, to regulate the formation of stomata (Hofmann, 2008). MUTE marks a subset of meristemoids that are differentiating into guard mother cells (Pillitteri et al., 2007). Additionally, *PME6* mRNA was highly accumulated in the *scrm-D* mutant, which has an excess of mature guard cells, and epidermal cell differentiation was blocked at the pavement cell stage (Amsbury et al., 2016). Transcriptome analysis revealed that *PME53* expression was also upregulated in *scrm-D mute*-double mutant plants (Pillitteri et al., 2011). Based on literatures, the present study focused on the analysis of *SCRM* and *MUTE* genes, which are strongly linked to pectin de-methylesterification of the guard cell wall. The results of the transactivation assay and RNA level highlighted the propensity of *PME53* to be positively regulated by SCRM and MUTE (Figure 6), indicating that PME53 may function as a critical element for stomatal development at a relatively late stage of differentiation.

Abscisic acid might be involved in stomatal initiation and differentiation by repressing SPCH and MUTE (Le et al., 2014), even though our data indicated that *PME53* was positively regulated by ABA and SCRM, as well as MUTE. SCRM is also an inducer of CBF expression 1 (ICE1), a master regulator of freezing tolerance, mediating the cold stress signal *via* an ABA-independent pathway (Kanaoka et al., 2008; Liang and Yang, 2015). To the current our knowledge, little is known about the heterodimerization between SCRM and MUTE is involved in ABA-regulated which still need to be investigated. With limited studies on the divergent regulation of ABA, how ABA-mediated stomatal development through the regulation of PME53 has yet to be determined.

### The Defective of *PME53* With Altered the Status of Pectin Methylation Has an Impaired Stomatal Function in Response to Heat Stress

Pectins have a conserved role in stomata, and the modification of the composition and structure of guard cell walls can directly affect stomatal functions. In the guard cell wall of *pme6*, methylesterified pectins are enriched, and the response to stomatal opening/closure triggers may be due to a mechanical change in the guard cell wall (Amsbury et al., 2016). The *STOMATAL CARPENTER1* (*SCAP1*) mutant, the pectin de-methylesterification in the ventral wall of guard cell walls was suppressed and showed an abnormal shape of stomata and reduced efficiency of stomatal movement (Negi et al., 2013). The degrees of de-methylesterified pectin on *pme53* and cotyledons differed from that of Col, indicating that their PME activity was affected (Figure 3A). Thus, this finding suggests that stomatal function is involved in the de-methylesterification of pectin in the guard cell wall. Our results may establish a link between the genes of guard cells and their cell wall properties with the corresponding effect of PME de-methylesterification on stomatal function.

During PMEs acting on the HGA chain, the effects can be classified as linear or non-block-wise de-methylesterification. The non-blockwise removal of methyl groups of HGA by PME following PG activity catalyzes the degradation of pectin and thus loosens the cell wall. In addition, it is hypothesized that the optimal PMEs at acidic pH tend to be randomly active in the HGA chain. AtPME2 participates in pectin de-methylesterification by randomly acting on the HGA chains, leading to the creation of substrates for PGs and consequent de-structuration of HGA; therefore, *pme2* showed high methylesterification of pectins, which prevented the hydrolysis by PGs and reduced elongation by stiffening the walls (Hocq et al., 2021). In this study, the activity of PME53 appeared to change with pH, indicating a pronounced pH dependence of its activity. PME53 exhibited optimum activity in the acidic context of the cell wall, suggesting that PME53 might mediate the random de-methylesterification of pectin (Figures 3C,D). That is, PME53 may contribute to loosening the cell wall by mediating the de-methylesterification of HGA randomly at acidic pH, leading to the formation of demethylated stretches. We suggest that the enzymatic activity of PME53 has dramatic consequences on cell wall texture and mechanical properties.

Apoplastic Ca^2+^ is essential for controlling cell integrity, cell wall cohesion, and plasma membrane permeability in plant cells. The recovery of heat shock-released Ca^2+^ is essential for acquiring thermoprotection to mitigate lethal heat injury in both soybean and rice seedlings (Wu and Jinn, 2010; Wu et al., 2010). EDTA treatment chelates the apoplastic Ca^2+^ and hence lowers the thermotolerance of *pme53* (Figure 9A). This suggests that the thermoprotection of *pme53* might be due to the different levels of extracellular Ca^2+^, which can be transported into the cell as stress-induced pathway signals. As a result, de-methylesterification caused by PMEs in the cell wall can directly impact the properties of pectin’s cell wall in terms of extracellular free Ca^2+^. During heat shock, the [Ca^2+^]_cyt_ signature changes by initiating biphasic elevation after 10 min in rice (*Oryza sativa*) (Wu et al., 2012). In *pme53*, the expression of *AtCaM3* which is a key member participating in the heat signal pathway (Zhang et al., 2009), was significantly reduced after 20 and 40 min of heat treatment (Figure 9B). At 37°C heat shock for 40 min, the expression of *HSP70* and *HSP101* is altered in *pme53* compared to Col (Figure 9C), indicating that *PME53* may coordinate with heat stress signaling.

Based on our previous studies (Wu and Jinn, 2010; Wu et al., 2010, 2012, 2017, 2018), the cleavage of apoplastic Ca^2+^ bridges between pectic carboxyl groups by the activity of PMEs is important for cell wall remodeling to retain cell integrity under heat stress. Accordingly, we propose that the Ca^2+^ distribution in cells of *pme53* may be altered during heat shock, and thereby the *AtCaM3* perception of Ca^2+^ signal was affected in *pme53*. Ca^2+^ bridging is less effective on a pectic domain when PME53 acts randomly as non-blockwise de-methylesterification; other PMEs may be activated/compensated to work on linearly promoting the formation of Ca^2+^-mediated cross-linking which may confer the less heat-sensitive phenotype in *pme53*. The genetic evidence showed that *PME53* is required for heat response; however, the underlying molecular mechanism of PME53 remains to be elucidated.

## Conclusion

**FIGURE 11:**
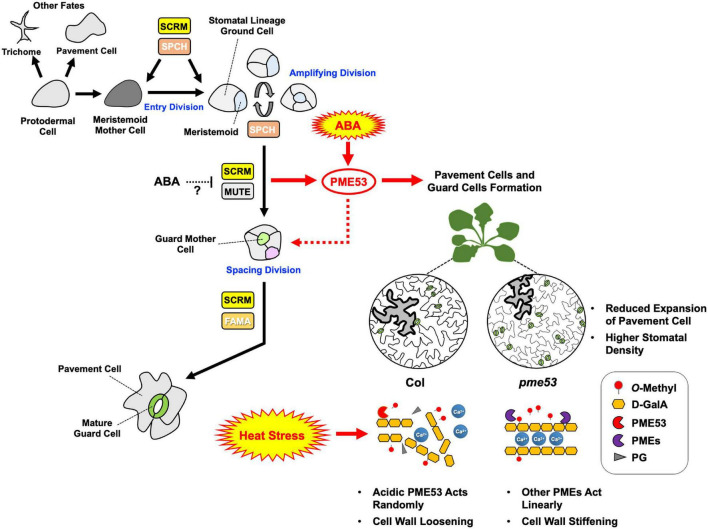
Arabidopsis *PME53*, a guard-cell specific gene, involves in stomatal development and stress response. The stomatal lineage consists of five major cell types: meristemoid mother cells (MMCs), meristemoids, stomatal lineage ground cells (SLGCs), guard mother cells (GMCs), and guard cells (GCs). GCs initiate from MMCs which are derived from a subset of protodermal cells. MMCs carry out asymmetric entry divisions to produce smaller meristemoids and larger SLGCs. Meristemoids undergo one to three rounds of asymmetric amplifying divisions before differentiating into GMCs. GMCs can undergo symmetric division to form a pair of GCs. Stomatal development is regulated by a group of bHLH transcription factors, including SPCH, MUTE, and FAMA, and the interacting factor SCRM, which are critical to mediate guard cell differentiation at different steps of stomatal lineage in Arabidopsis (Davies and Bergmann, 2014). PME53 may involve in stomatal cell lineage through the regulation of SCRM and MUTE transcription factors. ABA might be involved in stomatal development by repressing SPCH and MUTE. Whether the heterodimerization between SCRM and MUTE is involved in ABA-regulated, still need to be investigated. ABA may act upstream of *PME53* to regulate the function of PME53: a null mutation in *PME53* results in a significantly increased number of stomata as well as affected stomatal movement in response to heat as compared with the Col. During non-lethal heat stress, acidic PME53 acts randomly on pectin. Absence of PME53 may be led to active other PMEs which work on linearly promoting the formation of Ca^2+^ mediated cross-linking to confer the less heat-sensitive phenotype in *pme53*. Black arrows indicate that stomatal lineage signaling pathways have been thoroughly studied. Red arrows represent the new findings in this study, indicating that PME activity is governed by a network of signaling pathways related to ABA regulation and heat stress response.

To sum up our findings, we conclude with Figure 11. As demonstrated in this study, the expression levels of *PME53* were significantly affected through the expression of *SCRM* and *MUTE*, ABA signaling, and recovery from heat stress, therefore we propose that they likely act upstream of *PME53*, indicating that cell wall modifying enzymes are required to guard cell wall formation appropriately during normal stomatal development and heat response. But the action of ABA on the regulation of SCRM and MUTE interaction remains equivocal. The *pme53* exhibited a significantly high stomatal density, we suggest that is correlating with remarkable resistance to temperature elevation. Additionally, the role of PME53 combined with other PMEs in cell wall remodeling involved in Ca^2+^-pectate reconstitution may play a decisive role in thermotolerance to plants. Present evidence shows that the PME53-mediated de-methylesterification status of pectin is directed toward the effect on stomatal development, movements, and regulating the flexibility of the guard cell wall required for proper heat response.

## Data Availability Statement

The original contributions presented in the study are included in the article/Supplementary Material, further inquiries can be directed to the corresponding author/s.

## Author Contributions

All authors listed have made a substantial, direct, and intellectual contribution to the work, and approved it for publication.

## Conflict of Interest

The authors declare that the research was conducted in the absence of any commercial or financial relationships that could be construed as a potential conflict of interest.

## Publisher’s Note

All claims expressed in this article are solely those of the authors and do not necessarily represent those of their affiliated organizations, or those of the publisher, the editors and the reviewers. Any product that may be evaluated in this article, or claim that may be made by its manufacturer, is not guaranteed or endorsed by the publisher.

## Footnotes

1
http://www.cazy.org/

2
http://primer3.ut.ee/

3
http://wmd3.weigelworld.org/cgi-bin/webapp.cgi

4
https://imagej.nih.gov/ij/download.html

5
http://plantpan.itps.ncku.edu.tw/index.html

6
http://www.uniprot.org/
