# Supplementary material for: Guard Cell-Specific Pectin METHYLESTERASE53 Is Required for Abscisic Acid-Mediated Stomatal Function and Heat Response in Arabidopsis
Source: Front Plant Sci. 2022 Feb 21;13:836151. doi: 10.3389/fpls.2022.836151 (PMC8898962; doi:10.3389/fpls.2022.836151)
Supplement: Supplementary file 1 [file Data_Sheet_1.PDF]

Supplementary Table 1. Primers for genotyping, cloning, qPCR, and RT-PCR, as well as accession numbers of genes.

Gene (Purpose)	Primer	Sequence (5'→3')
PME53 (Genotyping)	LP	AACACGACCCCAGTC
	RP	TCATAAAGAGAAAGGGCATTTCC
T-DNA border (Genotyping)	BP	ATTTTGCCGATTTCGGAAC
PME53 (ORF)	Fw	AAGCTTATGCCCAAACTCAATTCAAC
	Rv	GGATTCGAGTTTGATCCATTCAGAGC
PME53 (Promoter)	Fw	AAGCTTAAACACTCACACCAAACATA
	Rv	GGATCCCTATACGACAATTAGCATCA
PME53 (q-PCR)	Fw	AGACCCTTCCCGTGAGATGA
	Rv	TGATCCATCAGAGCCGTCG
PP2A (q-PCR)	Fw	CCTGCGGTAATAACTGCATCT
	Rv	CTTCACTTAGCTCCACCAAGCA
RD29B (q-PCR)	Fw	AGTCGCCACGGTCCGTTGAAG
	Rv	CCGCCACTGCCTCCCAACTC
HSP18.1 (qPCR)	Fw	AGAACGATAAGTGGCACCGT
	Rv	ACCACAACCGTAAGCACACC
HSP70 (qPCR)	Fw	CCATCTGTCGGCATACCTCC
	Rv	AGAAGGCGATTGATGAAAC
HSP90 (qPCR)	Fw	GATCAACCCCGACAACGGTA
	Rv	AGCAGCAAAAGTGTTCGGTTC
HSP101 (qPCR)	Fw	TCACTTCTCTTTGGCCCGTT
	Rv	AAGATGGTTGTGCGTGAGGA
HSP18.1 (RT-PCR)	Fw	ACGTCTTTGATCCGTTCTCG
	Rv	ATTCATAACACAACAAGCCAAG
HSP70 (RT-PCR)	Fw	AGACAATCAACCAGGCGTTCT
	Rv	TCGCCTTCTCAATCTTCTGCT
HSP90 (RT-PCR)	Fw	GCGAGGTCTGGAACAAAAGAG
	Rv	ATCGGTTTCTGCTTGTTGATG
HSP101 (RT-PCR)	Fw	ACCGAGAAGAAGTCCTCTGGC
	Rv	TTTCCCACGAAGCTTCTCAAC
ACT2 (RT-PCR)	Fw	ATGAAGCACAATCCAAGAGAGGTA
	Rv	GAGCTTCTCCTTGATGTCTCTTACAA

UBQ10 (RT-PCR)	Fw	GATCTTTGCCGGAAAACAATTGGAGGATGGT
	Rv	CGACTTGTCATTAGAAAGAAAGAGATAACAGG
AtCaM3 (qPCR)	Fw	GGACTCGAGGTATGTTTTCTGCTT
	Rv	TGTTCAGACGCAAAATAGAGCATAA
SCRM (q-PCR)	Fw	AACACGACCCCAGTC
	Rv	TCATAAAGAGAAAGGGCATTTCC
MUTE (q-PCR)	Fw	CCAGACAATCGAGCCATCCA
	Rv	CCCACGATTCGCCTAGAGAC
SCRM1 (amiRNA)	Fw-I	GATTAAAGCCTATCATTAAGCTGTCTCTCTTTTGTATTCC
	Rv-II	GACAGCTTAATGATAGGCTTTAATCAAAGAGAATCAATGA
	Fw-III	GACAACTTAATGATACGCTTTATTCACAGGTCGTGATATG
	Rv-IV	GAATAAAGCGTATCATTAAGTTGTCTACATATATATTCCT
MUTE (amiRNA)	Fw-I	GATTTTTCGATTCGACTAAGCGGTCTCTCTTTTGTATTCC
	Rv-II	GACCGCTTAGTCGAATCGAAAAATCAAAGAGAATCAATGA
	Fw-III	GACCACTTAGTCGAAACGAAAATTCACAGGTCGTGATATG
	Rv-IV	GAATTTTCGTTTCGACTAAGTGGTCTACATATATATTCCT

Sequence data in this article can be found in the TAIR database under the following accession numbers:

HSP18.1 (At5g59720), HSP70 (At1g16030), HSP90 (At5g52640), HSP101 (At1g74310), PME34 (At3g49220), PME53 (At5g19730), RD29B (At5g52300), AtCaM3 (At3g56800), SCRM (ICE1; At3g26744), MUTE (At3g06120).

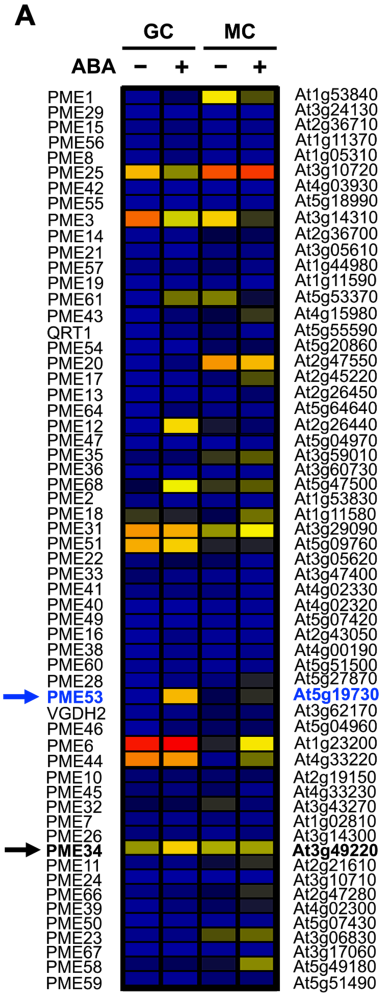

B

AGI Code		Gene Name	At5g19730	At3g49220
Affy ID		Probe Set Name	245965_at	252255_at
		Common Name	***PME53***	***PME34***
With Transcriptional Inhibitors	Guard Cells	Signal	0.00	364.95
	Guard Cells + ABA	Signal	881.96	617.26
	Mesophyll Cells	Signal	148.29	398.01
	Mesophyll Cells + ABA	Signal	219.82	384.21

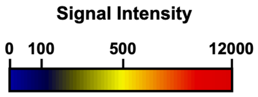

Supplementary Figure 1. Transcription profiling of *PME* genes in guard cells (GC) and mesophyll cells (MC) in response to ABA treatment. (A) Affymetrix Arabidopsis ATH1 microarray data retrieved from the MIAMExpress (http://ebi.ac.uk/miamexpress) with the accession number E-MEXP-1443 (Yang et al., 2008). Putative *PME* genes were selected for the indicated genes and were derived as described by Pelloux et al., (2007). Gene hierarchical clustering analyses were performed with GeneSpring 7.3 (Silicon Genetics) using Pearson correlation. The color code of signal intensities corresponds to the abundance of transcripts as indicated range from blue (low expression) to red (high expression). **(B)** Transcriptional profiles of *PME53* gene in both guard cells and mesophyll cells in response to ABA treatment. *PME34* was used as a reference. Cells were treated without (−) or with (+) 100 μM ABA for 1 h.

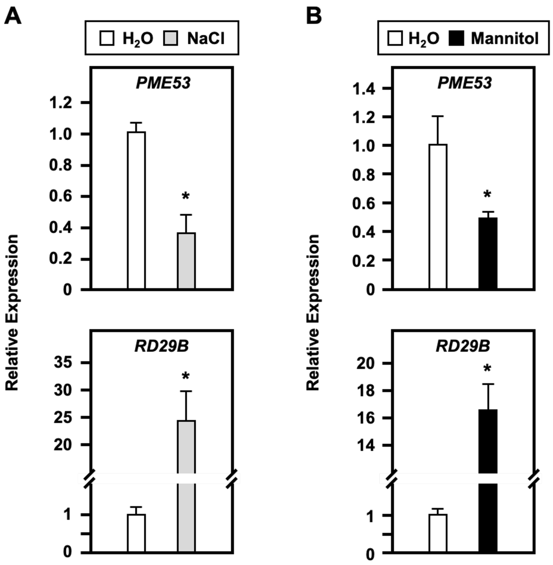

Supplementary Figure 2. Transcriptional level of *PME53* in response to abiotic stress. (A) and **(B)** 7-d-old seedlings were treated with 150 mM NaCl for 6 h and 300 mM mannitol for 6 h, respectively, and then the expression levels of *PME53* were analyzed by q-PCR. *RD29B*, a salt and ABA-responsive gene, was used reference. The fold change expression was normalized relative to level of the H_2O control. Data are mean ± SE of three biological replicates. *, Significant at $P < 0.05$ compared with the H_2O treatment.

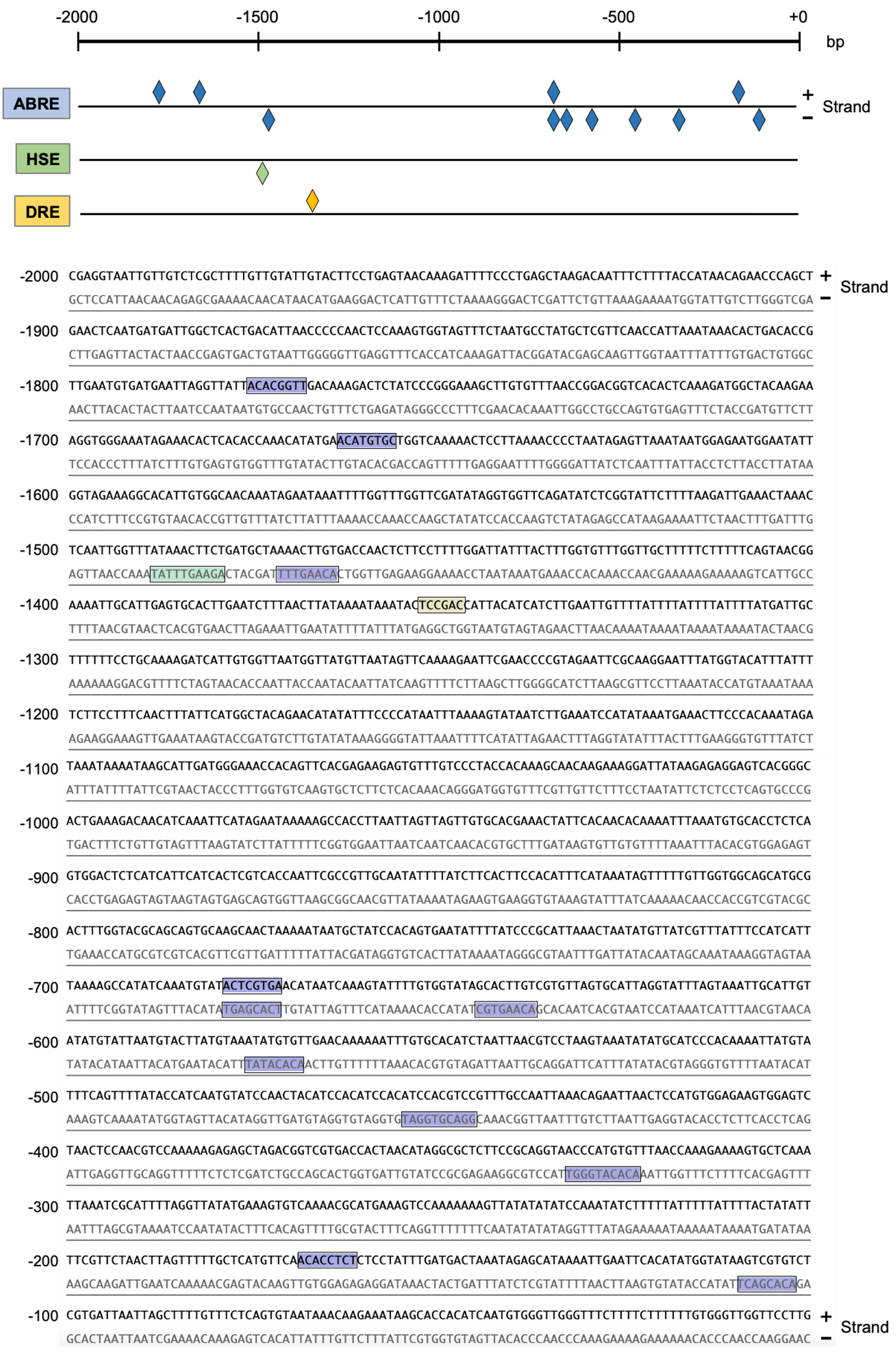

Supplementary Figure 3. Potential *cis*-elements in 2 kb promoter region of *PME53*. The ABREs, DREs, MYBs, and MYCs elements were characterized with The Plant Promoter Analysis Navigator, a database for transcription regulatory networks[5].

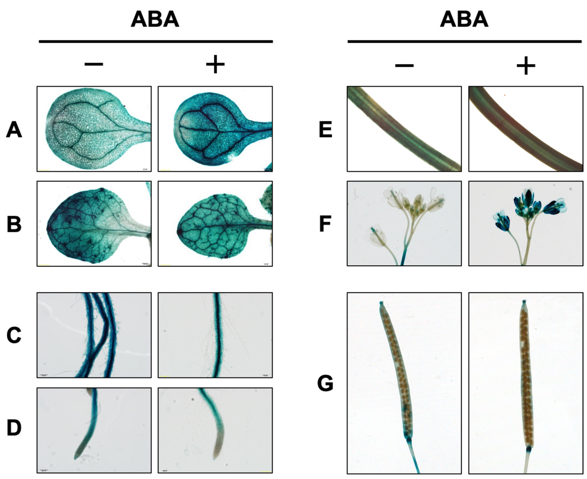

Supplementary Figure 4. Histochemical analysis of *PME53*-promoter::*GUS* expression. GUS staining in tissues were treated without (−) or with (+) 30 μM ABA for 3 h, in 12-d-old cotyledon **(A)**, rosette leaf **(B)**, root mature zone **(C)**, and root tip **(D)**, as well as 4-week-old florescence stem **(E)**, flowers **(F)**, and silique **(G)**.

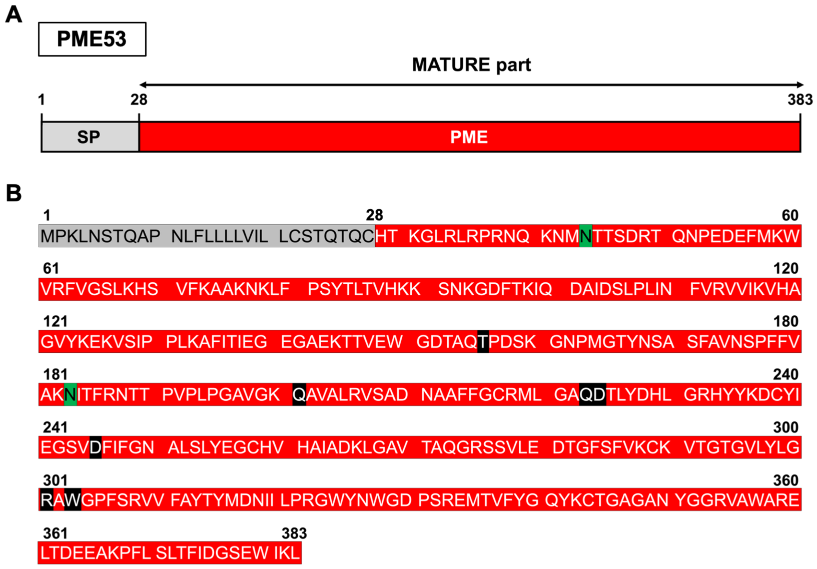

Supplementary Figure 5. PME53 protein structure motifs and amino acid sequence. (A) The structural motifs of a type-II PME, PME53 (UniPro: Q8VYZ3). Preceding is a conserved PME domain (amino acids 29 to 383; highlighted with red). As well, the signal peptide (SP; highlighted with gray). **(B)** Amino acids involved in the matured-PME potential catalytic site (residues of T166, Q201, Q223, D224, D245, R301, W303; highlighted with black) and glycosylation site (two Asp residues; highlighted with green) are indicated.

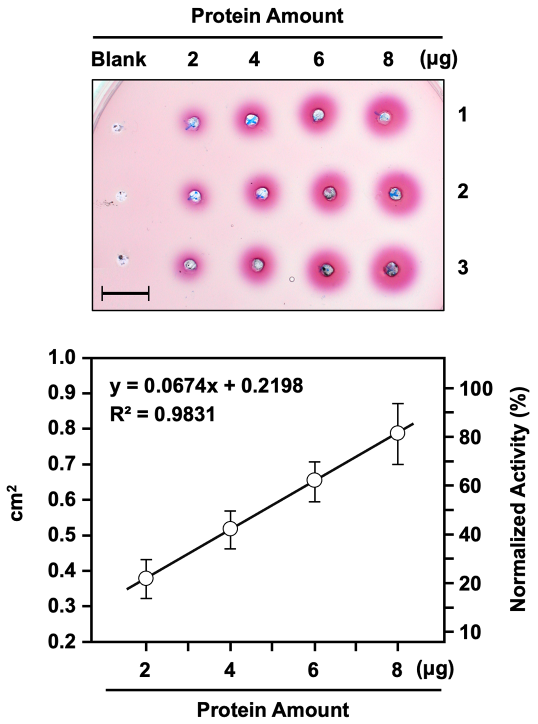

Supplementary Figure 6. A gel diffusion assay for quantification of PME activity. The standard linearity curve of PME activity was obtained at pH 6 by using different amounts of cell-wall protein input. The ruthenium red (RR) dye-stained zone diameters resulting from the hydrolysis of esterified pectin in an agarose gel were measured. The R^2 value for the linear regression is at 0.9831. Data are mean ± SE of three biological replicates.

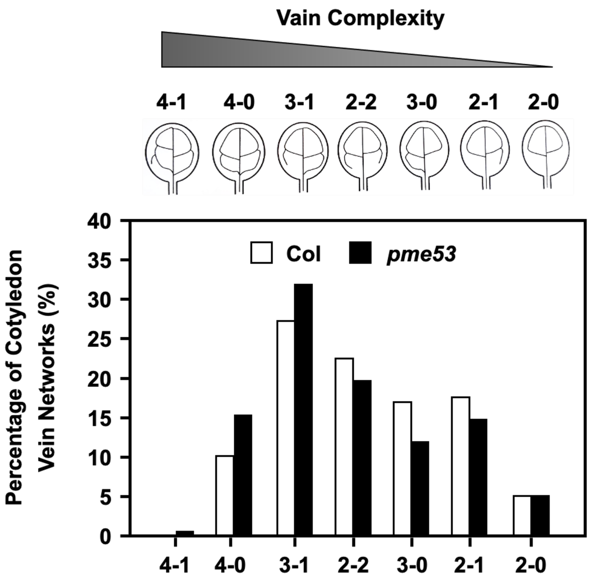

Supplementary Figure 7. Vein networks in Col and *pme53* muatnt. Cotyledon vein networks in 7-d-old cotyledons were analyzed. Number of closed areoles (2, 3, or 4) formed by the secondary veins and the number of vein branches/incomplete areoles in the proximal (closest to the petiole) part of the cotyledon. From left to right is shown the high to low complexity of vein networks **(top)**. The vein complexity patterns (%) of cotyledon from Col and *pme53* were analyzed **(bottom)**. n = 50 cotyledons.

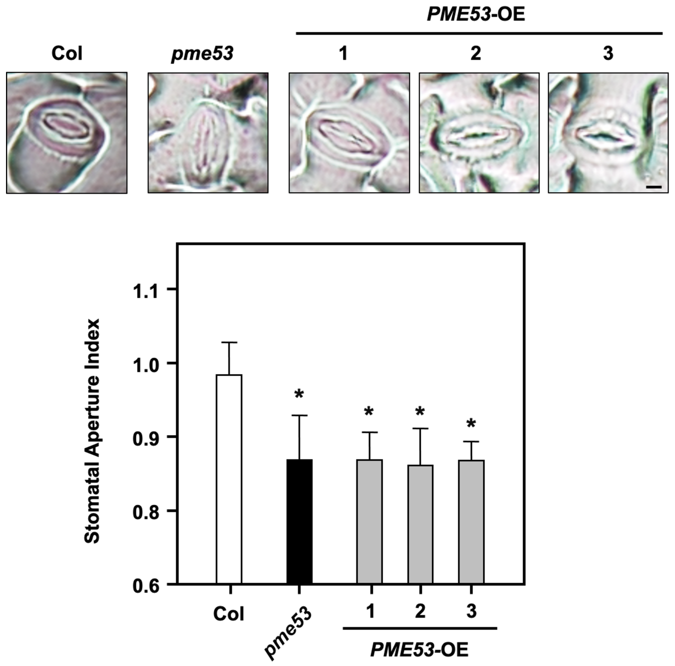

Supplementary Figure 8. Stomata phenotype in Col, *pme53* mutant and three *PME53*-OE lines. The stomata on the abaxial surface of 3rd and 4th pair leaves of 4-week-old plants was was photographed **(top)** and mensured **(bottom)**. The stomatal aperture index compared with the Col, as indicated in Figure 5. Data are mean ± SE of three biological replicates. *, significant at $P < 0.05$ compared with the Col. $n > 80$ stomata. Silicon polymer polymer molds were taken from the surface of individual leaves at a similar region of testers and filled with epoxy resin. The obtained replicas were sputter coated and observed by scanning electron microscopy as described by Huang et al. (2017).

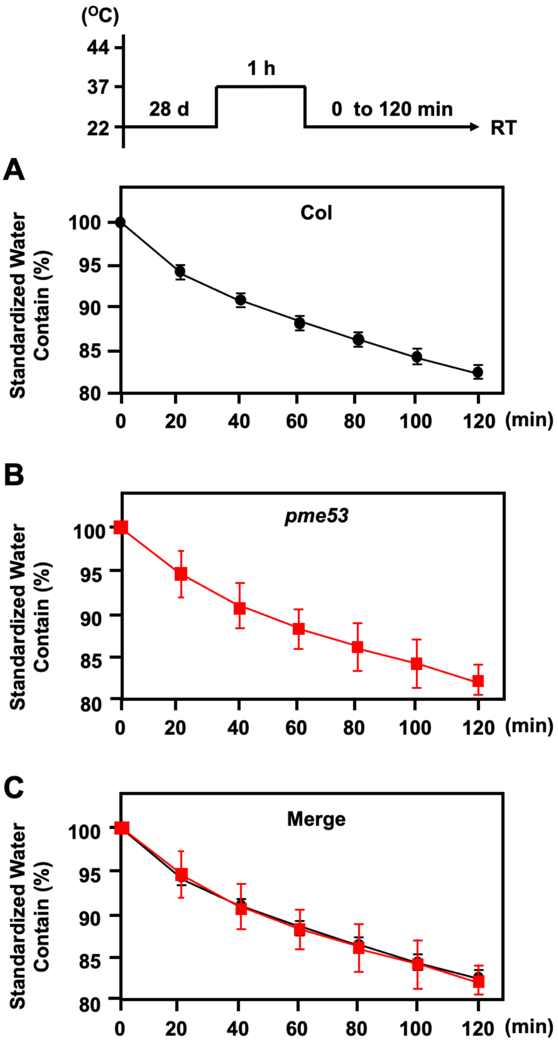

Supplementary Figure 9. Transpiration rate in Col and *pme53* mutant after mild heat shock treatment. The detached leaves of 4-week-old plants of Col and *pme53* were treated with 37°C for 1 h, then the standardized water loss (%) was measured. Data are mean ± SE of five biological replicates. n = 40 leaves.

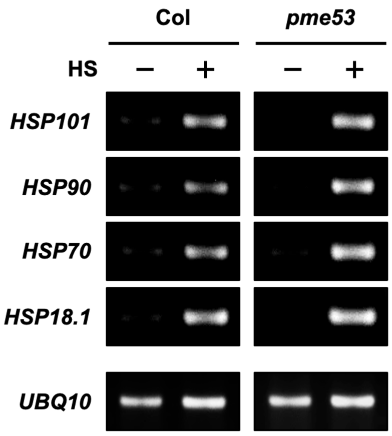

Supplementary Figure 10. Transcriptional levels of major heat shock-responsive genes in Col and *pme53* mutant in response to heat stress. 7-d-old seedlings were treated without (−) or with (+) heat at 37°C for 1 h (HS). The expression levels of the heat stress up-regulated marker genes *HSP101, HSP90, HSP70*, and *HSP18.1* were analyzed by RT-PCR. *Ubiquitin10* (*UBQ10*) was used as loading control.

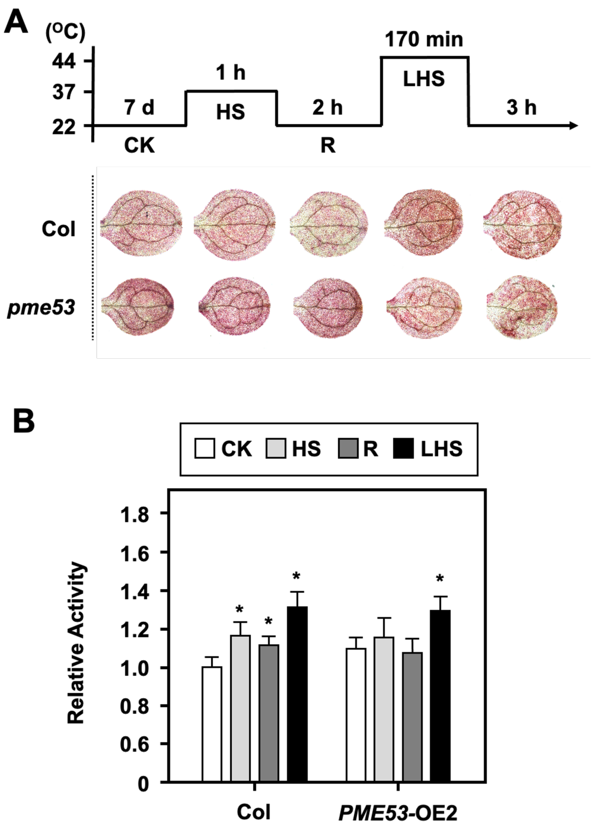

Supplementary Figure 11. Levels of de-methylesterified pectins in Col and *pme53* mutant and PME activity in *PME-OE2* plants in response to heat stress. The pictogram shows the heat stress regime. **(A)** 7-d-old cotyledons stained with the ruthenium red (RR) were photographed. RR stained with demethylesterified pectin in different degrees from brown (less demethylesterified) to red (more demethylesterified). **(B)** The PME activity in 7-d-old seedlings were analyzed as described in Figure 8. The fold change PME activity was normalized relative to level of the control (CK). Data are mean ± SE of three biological replicates. *, significant at $P < 0.05$ compared with the control treatment.
